# Stromal vascular fraction promotes migration of fibroblasts and angiogenesis through regulation of extracellular matrix in the skin wound healing process

**DOI:** 10.1186/s13287-019-1415-6

**Published:** 2019-10-17

**Authors:** Hongsen Bi, Hui Li, Chen Zhang, Yiqing Mao, Fangfei Nie, Ying Xing, Wuga Sha, Xi Wang, David M. Irwin, Huanran Tan

**Affiliations:** 1 Department of Plastic Surgery, Peking University Third Hospital, Beijing, 100191 China; 2 Department of Pharmacology, Peking University, Health Science Center, Beijing, 100191 China; 3 Department of Laboratory Medicine and Pathobiology, University of Toronto, Toronto, Ontario M5S1A8 Canada

**Keywords:** Stromal vascular fraction, Human adipose-derived stem cells, Wound healing

## Abstract

**Background:**

A refractory wound is a typical complication of diabetes and is a common outcome after surgery. Current approaches have difficulty in improving wound healing. Recently, non-expanded stromal vascular fraction (SVF), which is derived from mature fat, has opened up new directions for the treatment of refractory wound healing. The aim of the current study is to systematically investigate the impact of SVF on wound healing, including the rate and characteristics of wound healing, ability of fibroblasts to migrate, and blood transport reconstruction, with a special emphasis on their precise molecular mechanisms.

**Methods:**

SVF was isolated by digestion, followed by filtration and centrifugation, and then validated by immunocytochemistry, a MTS proliferation assay and multilineage potential analysis. A wound model was generated by creating 6-mm-diameter wounds, which include a full skin defect, on the backs of streptozocin-induced hyperglycemic mice. SVF or human adipose-derived stem cell (hADSC) suspensions were subcutaneously injected, and the wounds were characterized over a 9-day period by photography and measurements. A scratch test was used to determine whether changes in the migratory ability of fibroblasts occurred after co-culture with hADSCs. Angiogenesis was observed with human umbilical vein endothelial cells. mRNA from fibroblasts, endotheliocyte, and skin tissue were sequenced by high-throughput RNAseq, and differentially expressed genes, and pathways, potentially regulated by SVF or hADSCs were bioinformatically analyzed.

**Results:**

Our data show that hADSCs have multiple characteristics of MSC. SVF and hADSCs significantly improved wound healing in hyperglycemic mice. hADSCs improve the migratory ability of fibroblasts and capillary structure formation in HUVECs. SVF promotes wound healing by focusing on angiogenesis and matrix remodeling.

**Conclusions:**

Both SVF and hADSCs improve the function of fibroblast and endothelial cells, regulate gene expression, and promote skin healing. Various mechanisms likely are involved, including migration of fibroblasts, tubulogenesis of endothelial cells through regulation of cell adhesion, and cytokine pathways.

## Background

Diabetes is a serious metabolic disease that threatens human health and results in a huge economic burden for China [1]. Vascular lesions are the pathological basis for many of the chronic complications of diabetes, including major vascular lesions and microvascular lesions [2]. In diabetic patients, large vascular lesions can cause ischemic changes in the skin, leading to ulcers, infections, and wounds that do not heal [3]. The refractory wound is a typical complication of diabetes and is also a common occurrence after surgery [4]. These wounds are characterized by having many complications that yield a large influence on their appearance [5]. Serious cases can lead to amputation. Effective control and treatment of diabetic wound healing can significantly reduce the risk of amputation, reduce medical expenses, and improve the quality of the life of patients [6, 7]. However, to date, there is no ideal treatment; therefore, improving the healing of refractory wounds is of great clinical value and significance.

Skin wound healing is a complex biological integration process and includes the aggregation of extracellular matrix molecules, soluble media and cytokines, proliferation and migration of multiple cells, extracellular matrix deposition, and reconstruction of blood transport systems [8]. However, in diabetics, the orderly integration of these processes is disrupted [9]. At present, conventional clinical treatment methods include removal of the trauma, local wound medication, and some newer treatment methods such as electrical stimulation, treatment with growth factor, and negative pressure treatment [10]. These methods have had only moderate clinical efficacy in improving chronic wound healing [11, 12]. Recently, stem cells, especially adipose-derived stem cells (ADSCs) that have multidirectional differentiation potential and an ability to secrete various growth factors and inflammation factors [13, 14], have become important tools in transplantation therapy to promote the healing of refractory wounds [15]. Several research teams have shown that ADSCs can effectively promote the healing of refractory wounds [16, 17]. However, isolation of ADSCs requires in vitro cell culture and expansion and can take from days to weeks. These steps increase the possibility of infection by microorganisms, such as bacteria, and thus, can be difficult for the clinical application of ADSC-based cytotherapy [18]. At the same time, studies have found that non-expanded stromal vascular fraction (SVF) that is derived from digestion, filtration, and centrifugation of mature fat cells contains a variety of fat cells and mesenchymal stem cells (MSCs) that opens up new directions for the treatment of difficult wounds [19].

Although SVFs are helpful for the treatment of many diseases, currently it is not commonly used in the clinic [20]. Despite extensive research, the precise mechanisms used by SVF to promote wound healing are still unclear, which impedes the progress in the application of SVF cells. The aim of our study is to determine whether therapy with non-expanded cell SVF is as efficient as expanded ADSCs in the treatment of wounds and to evaluate the therapeutic potential of autologous SVF. We systematically investigated the effects of SVF and hADSCs on wound healing, including the rate of wound healing, characteristics of the newly generated epithelium, effect on the migration ability of fibroblasts, and blood transport system reconstruction, with a special emphasis on the precise mechanisms. The results provide a new experimental basis for further understanding mechanisms used by SVF and support promoting the clinical application of these cells.

## Materials and methods

### Ethical approvals

All experiments were performed in accordance with the guidelines and study protocols of the Peking University Third Hospital Medical Science Research Ethics Committee (Approval No. IRB00006761-M2017391).

### Harvesting of fat and SVF cell preparation

Human adipose tissue was obtained from male and female diabetic patients by a casing needle (fat suction) after informed consent and approval of the Peking University Third Hospital Medical Science Research Ethics Committee. After fat suction, adipose tissue was digested with 0.1% collagenase I (Gibco, USA, 17100017) and then centrifuged at 1500 rpm for 5 min to obtain a cell pellet. After filtration through a 425-μm mesh (Solarbio, CHN), the SVF cells were counted. Some of the SVFs were immediately used in animal experiments, with the remaining cells cultured. After 2 days of culture, media was changed to remove non-adherent cells while the attached cells were continued to be cultured in DMEM (Gibco, USA, 12800017) with 10% FBS (Bioind, Israel, 04-001-1ACS). After the cell colonies reached 80% confluence, cells were digested with trypsin. These cells were designated the first passage of human adipose-derived stem cells (hADSCs) [21]. In this study, we used cells from the third to the fifth passage.

### Immunocytochemical characterization of SVF and hADSCs

Unamplified SVF cells and pure adherent homogenous populations of cells obtained after culturing the SVF cells were characterized for the presence or absence of MSC marker proteins. After fixing with 4% polyformaldehyde and incubation with 5% BSA (Amersco, USA, A0332), cells were then incubated with mouse antibodies to CD105, CD44, and CD90 (each 1:100) and with rabbit antibodies to CD29 (1:100) and CD45 (1:200) (Abcam, USA, Mesenchymal Stromal Cell Marker Antibody Panel (CD44, CD45, CD90, CD29, CD105) - Human, ab93758) overnight at 4 °C. Antibody binding was detected using goat anti-mouse antibodies conjugated to FITC (Zsbio, CHN, ZF-0312) or goat anti-rabbit antibodies conjugated to TRITC (Zsbio, CHN, ZF-0316) by incubating in the dark for 1 h. The nuclei of cells were stained with 300 nM DAPI (Solarbio, CHN, C0065), and fluorescence was observed through a confocal microscope (Leica, Germany).

### Assays for adipogenic and osteogenic differentiation potential

Assays for adipogenic differentiation potential was performed according to the manufacturer’s instructions (Cyagen, CHN, HUXMD-90031). Briefly, hADSCs were seeded into six-well plates, with human fibroblast (hF) cells used as control. When the cells reached 80% confluence, the media was replaced with adipogenic differentiation medium A (Cyagen, CHN, HUXMD-90031). After using medium A for 3 days, medium B (Cyagen, CHN, HUXMD-90031) was replaced for 24 h. After using medium A and B alternately for five times, medium B was used continuously for 7 days until the fat drops become large and round enough. During the culture period of medium B, fresh medium B was replaced every 3 days. After about 27 days of culture, cells were stained with Oil Red O according to the following method. Cells were fixed with 50% ethanol for 3 min and then dyed with 5% Oil Red O for 30 min at room temperature. Induced fat cells contain orange-red oil droplets.

For assays for osteogenic differentiation potential, as with the adipogenic differentiation experiments, media was replaced with osteogenic differentiation medium (Cyagen, CHN, HUXMD-90021) when confluence reached 70% in the six-well plates. Osteogenic media was changed every 3 days. After about 21 days of culture, Alizarin red S staining was performed. Cells were fixed with 50% ethanol for 3 min, and then stained with 1% Alizarin red S for 10 min at room temperature. Induced osteoblast cells contain red crystal particles.

### Healing of full-thickness wounds in diabetic mice

Male C57 mice (4–6 weeks old) were purchased from the Animal Center of the Peking University Health Science Center (Beijing, CHN). Animals were housed under controlled environmental conditions, including temperature, humidity, and lighting. Standard laboratory chow and water were provided ad libitum. A protocol for these experiments, following the “Guidelines for Animal Experiments,” was approved by the Peking University Health Science Center. C57 mice were randomly assigned to the following two groups: blank (normal saline, intraperitoneal injection) and STZ (streptozocin 200 mg/kg, intraperitoneal injection) (Coolaber, CHN, CS10491). Fasting serum glucose was measured 7 days after treatment with a Roche blood glucose monitor (Roche, Germany).

**Fig. 1:**
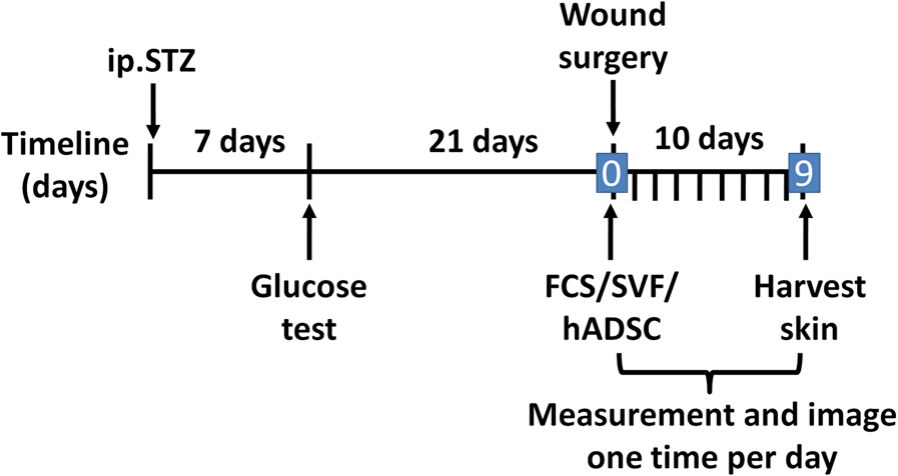
Timeline for the experimental design. Initiation of treatment with SVF or hADSCs was considered to be day 0 (indicated in the blue box), with wounds photographed and measured each day until day 9

Hyperglycemic mice were raised for an additional 21 days, and then full-thickness wounds were created. Under general anesthesia, hair in the surgical area was removed and a full-thickness excisional wound of 6 mm diameter was produced on the back of each mouse. To prevent the wound from spontaneously contracting, a plastic ring was placed in the wound. Hyperglycemic mice (*n* = 19) were divided into three groups: SVF treated (SVF, injection of SVF suspension with FBS into the four quadrants, *n* = 5), hADSC treated (hADSC, injection of hADSC suspension with FBS into the four quadrants, *n* = 6), and control (CON, injection of FBS, *n* = 8). Local injections of cell suspensions contained 1.5 × 10^5^ cells. On the day of the operation and every day thereafter, the state of the wound was recorded by photography. Nine days after the operation, a 2-mm-wide slice of skin from around the wound surface was harvested, with half of each sample paraffin-embedded for HE staining and the remainder frozen in liquid nitrogen and stored at − 80 °C for RNA analysis. Figure 1 describes the experimental design used in this study.

The long and short diameters of the wounds were measured with a cursor caliper, and the area was calculated using the following formula:

$$ \mathrm{Area}=\pi \times \mathrm{long}\ \mathrm{diameters}\times \mathrm{short}\ \mathrm{diameters}. $$

### Histology and immunohistochemistry

For HE and Masson staining, 4-μm-thick paraffin-embedded sample slices were prepared. These experiments were performed by Servicebio (CHN). Images were taken using an Olympus1X71 microscope (Olympus, JPN). Image-Pro Plus 6 (Media Cybernetics, USA) was used to measure the thickness of the epidermis and evaluate the integrity of the new skin in the HE staining. Collagen was quantified by calculating the ratio of the positive area (blue–green collagen) to the total area in the skin tissue Masson staining.

In order to detect the presence of CD31, immunohistochemistry was carried out. Rehydrated paraffin sections were incubated in a microwave oven for 8 min. All sections were blocked with 3% BSA at 37 °C for 30 min, followed by incubation with anti-CD31 antibody (Servicebio, GB11063-3, 1:800) at 4 °C overnight. After washing with PBS, a goat anti-rabbit secondary antibody (Servicebio, GB23303, 1:200) coupled with horseradish peroxidase was employed. Samples were incubated at 37 °C for 1 h. Sections were visualized with diaminobenzidine tetrahydrochloride, and staining was detected with a light microscope (Olympus, JPN). CD31 levels were quantified by calculating the ratio of the positive area (yellowish-brown) to the total area using Image-Pro Plus 6 (Media Cybernetics, USA).

### MTS proliferation assay

hADSCs (2 × 10^3^ per well) were seeded into 96-well plates, and then formazan production was determined using the CellTiter 96 AQueous Non-Radioactive Cell Proliferation Assay (Promega, USA, G3582) according to the manufacturer’s instructions. Dehydrogenase produced by living cells can convert MTS to formazan, with formazan having an absorption peak at 490 nm. OD value at 490 nm reflects the ability of cells to proliferate.

### Migration ability assay

A scratch test was used to determine changes in the migratory ability of fibroblast cells in co-culture with hADSCs. Primary human fibroblasts (hFs) were cultured from normal human skin. hADSC or hF cell suspensions that had been continuously cultured for 4–8 passages were seeded (1 × 10^4^ cells) into the upper chamber of 8-mm pore size 24-well transwell plates (Corning, USA), with 5 × 10^4^ hFs cultured in the lower chamber. Transwell plates were cultured for 24 h with DMEM full culture medium, with cells attaining 90% confluence. A scratch model was prepared by drawing a straight line on the surface of the lower chamber with a 200-μl pipette tip, with PBS used to wash off any exfoliated cells. Adherent cells were continued to be co-cultured with hADSCs or hFs for 0, 6, 12, 24, 36, 48, 60, and 72 h, and the scratch width was observed and analyzed with Image-Pro Plus 6 (Media Cybernetics, USA). Change in scratch width (ΔScratch) was calculated using the following formula:

$$ {\Delta \mathrm{Scratch}\ \mathrm{width}}_{\mathrm{n}}={\mathrm{SW}}_{\mathrm{i}}-{\mathrm{SW}}_{\mathrm{n}} $$

where SW_i_ represents the initial scratch width and SW_n_ is the scratch width at the *n*th hour after scratch formation.

### Tube formation assay

Human umbilical vein endothelial cells (HUVECs) (iCell Bioscience, CHN) were cultured in PriMed-iCELL-002 (iCell Bioscience, CHN) supplemented with 5% FBS. Subsequent experiments were conducted using cells at passage 2 to 4. Culture plates were pre-cooled to − 20 °C before the experiments. Matrigel (Becton, Dickinson & Co., USA, 356230) was laid on the bottom of the lower transwell chamber, with the chamber then placed at 37 °C for 2 h for solidification of the matrigel. HUVECs (1 × 10^5^/well) were seeded into the lower chamber. hADSCs or HUVECs (as a control) (1 × 10^4^/well) were plated into the upper chamber of the transwell, followed by incubation for 24 h before the experiments. Angiogenesis (tube formation) was observed and photographed after 0, 2, 4, 6, 8, and 10 h. The number of tube structures was counted using an Image-Pro Plus 6 (Media Cybernetics, USA).

### mRNA preparation for RNAseq

We used the transwell assay as a non-contacting co-culture system to evaluate the impact of hADSCs on fibroblast (3T3 cells) and endotheliocyte (MS1 cells). Fibroblast and endotheliocyte cells were seeded into the lower chamber of the transwell with hADSCs in the upper chamber. Cells were co-cultured for 72 h at 37 °C. Cells in the lower chamber were washed by PBS, and total RNA was isolated using Trizol LS reagent (Invitrogen, USA, 10296-010) following the instructions of the manufacturer.

Skin around the wounds was harvested at the end of the wound healing experiment. One half of each sample was snap-frozen, and total RNA was isolated using Trizol LS reagent (Invitrogen, USA, 10296-010) following the instructions of the manufacturer.

mRNA was purified from the 3 μg of total RNA per sample (cells or skin tissue) using poly-T oligo-attached magnetic beads, and then RNAseq was performed by Majorbio (Shanghai, CHN). Clean high-quality data obtained after removing reads containing ploy-N, reads containing adapters, and low-quality reads from the raw data was used for the subsequent analysis. DEGSeq R package (1.12.0) was used for the differential expression analysis. In this study, log2 (fold change) of > 1.00 and a corrected *P* value < 0.05 (adjusted using the Benjamini and Hochberg method) were necessary to classify a differentially expressed gene. Data were analyzed on the free online Majorbio I-Sanger Cloud Platform (www.i-sanger.com).

Significantly enriched KEGG and GO terms for differentially expressed genes were those with corrected *P* values less than 0.05. A protein-protein interaction (PPI) network of the differential genes was constructed using the STRING database (http://string-db.org/) and was visualized using Cytoscape for further analysis.

### Gene expression analysis using quantitative reverse transcription polymerase chain reaction (qRT-PCR)

Cell or skin tissue samples were homogenized, and total RNA was isolated using Trizol LS reagent (Invitrogen, USA, 10296-010) following the instructions. Amplifications were performed with the StepOne™ System with PowerUpTM SYBR® Green MasterMix (ABI, USA, 4367659) with the primers listed in Table 1. The amplification steps are as follows: initial denaturation at 95 °C for 10 min, followed by 40 cycles at 95 °C for 15 s and 60 °C for 1 min. *GAPDH* was used as an internal reference.

**Table 1:** Primers for quantitative reverse transcription polymerase chain reaction (qRT-PCR)

Gene name	Primer sequence (5′–3′)	
	Forward	Reverse
*edn1*	CGTCGTACCGTATGGACTGG	GCCTGGTCTGTGGCCTTATT
*lmod1*	ATGGTTGGACAGTGCCTGAG	TGGCATCAACCCTTCAGGTC
*Osm*	AGAATCAGGCGAACCTCACG	GTGTGTCCTCACTGGGGAAG
*Ccl4*	AGTCATCATTGAACAGGCTTTCT	CGAGTGCTGTGCTGATCTGA
*Ccr1*	ACTCTGGAAACACAGACTCACT	TGATGTGCTGCTGCGAGATT
*Col1a1*	ACAGTCGCTTCACCTACAGC	GGGTGGAGGGAGTTTACACG
*Col4a2*	CTGGTGAAGCACAGCCAAAC	GTCACCCGGATTGCAGTACA
*Itgb4*	CACGGACACCGGGATTATGT	AGACTCCTGTCCGTTTCATCG
*Sdc1*	CCTAACGCAGAGGAAGGGAC	TTCGTGGATCCGAGTTGCC
*Thbs1*	GCTGCCAATCATAACCAGCG	TTCGTTAAAGGCCGAGTGCT
*Mmp9*	CCAGCCGACTTTTGTGGTCT	TGGCCTTTAGTGTCTGGCTG
*Vipr1*	GCCTCCACACAAGGCAAATG	GTGTTTCCAGGTAGGGCACA
*Angptl7*	TCACGGGGAAGGAAGAAAACT	GCCTGTTCCCCTTAGAGTCTG
*GAPDH*	ACAAAGTGGACATTGTTGCC	AAACATGGTGGTGAAGACGC

### Statistical analysis

Results are shown as means ± SD. Differences between groups were analyzed by one-way ANOVA (SPSS 13.0 for Windows, SPSS Inc., USA). *P* values of less than 0.05 were considered to be statistically significant.

## Results

### Characterization of SVF surface markers

**Fig. 2:**
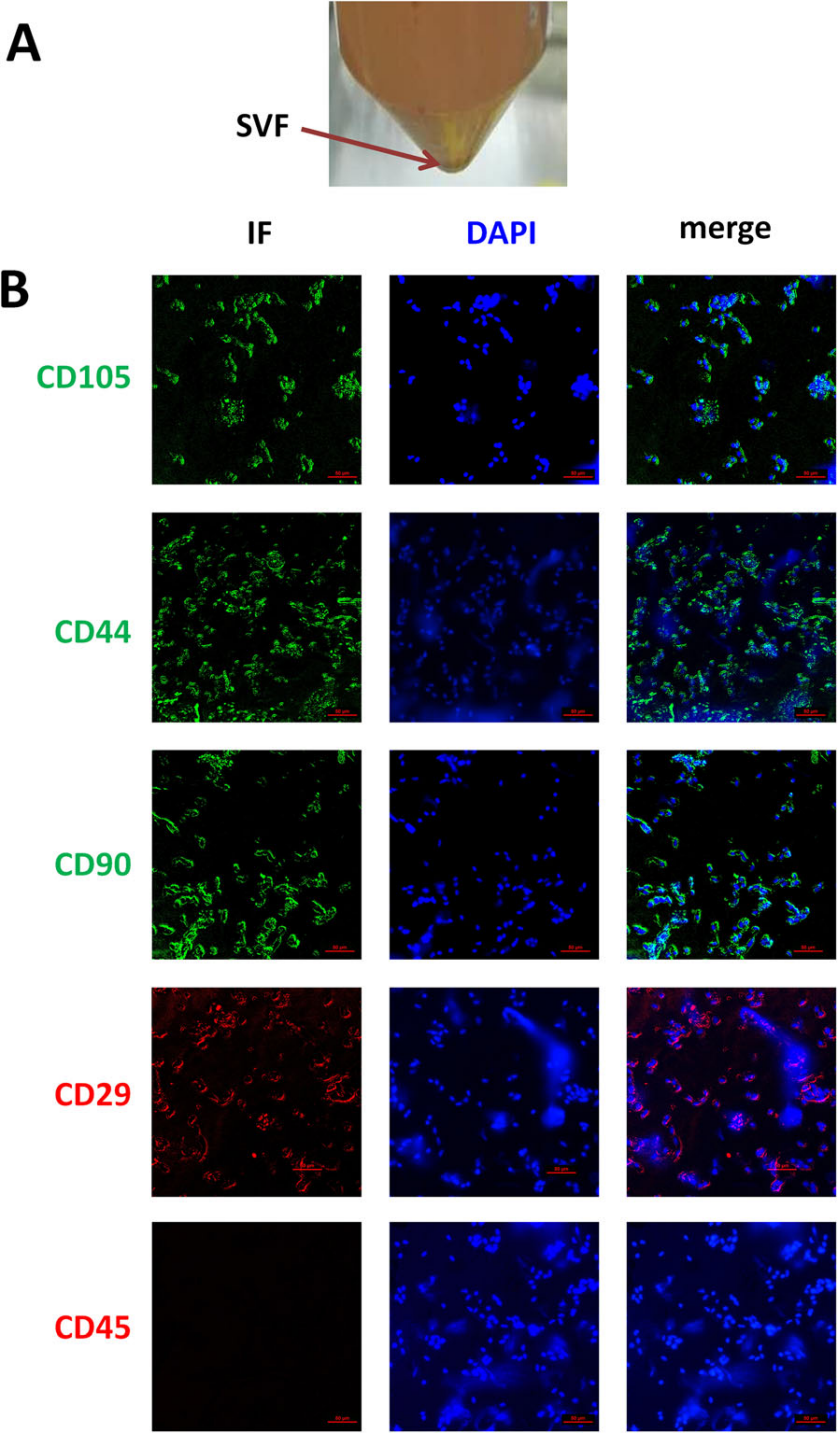
SVF surface marker characteristics. **a** After digestion and centrifugation of fat suction, obtained SVF (red arrow). **b** Immunofluorescence staining of CD105 (green), CD44 (green), CD90 (green), CD29 (red), and CD45 (red) in SVF, counterstained with DAPI (blue)

Adipose-derived SVF (Fig. 2a) are a heterogeneous population of cells. According to the International Society for Cellular Therapy position statement, MSCs are positive for CD105, CD44, CD90, and CD29 and have low expression of the negative marker CD45 [22]. SVF cells were characterized for these markers by immunocytochemistry, with our results confirming that the main cell type in the SVF population is hADSC (Fig. 2b).

### HADSCs share multiple characteristics with MSCs

**Fig. 3:**
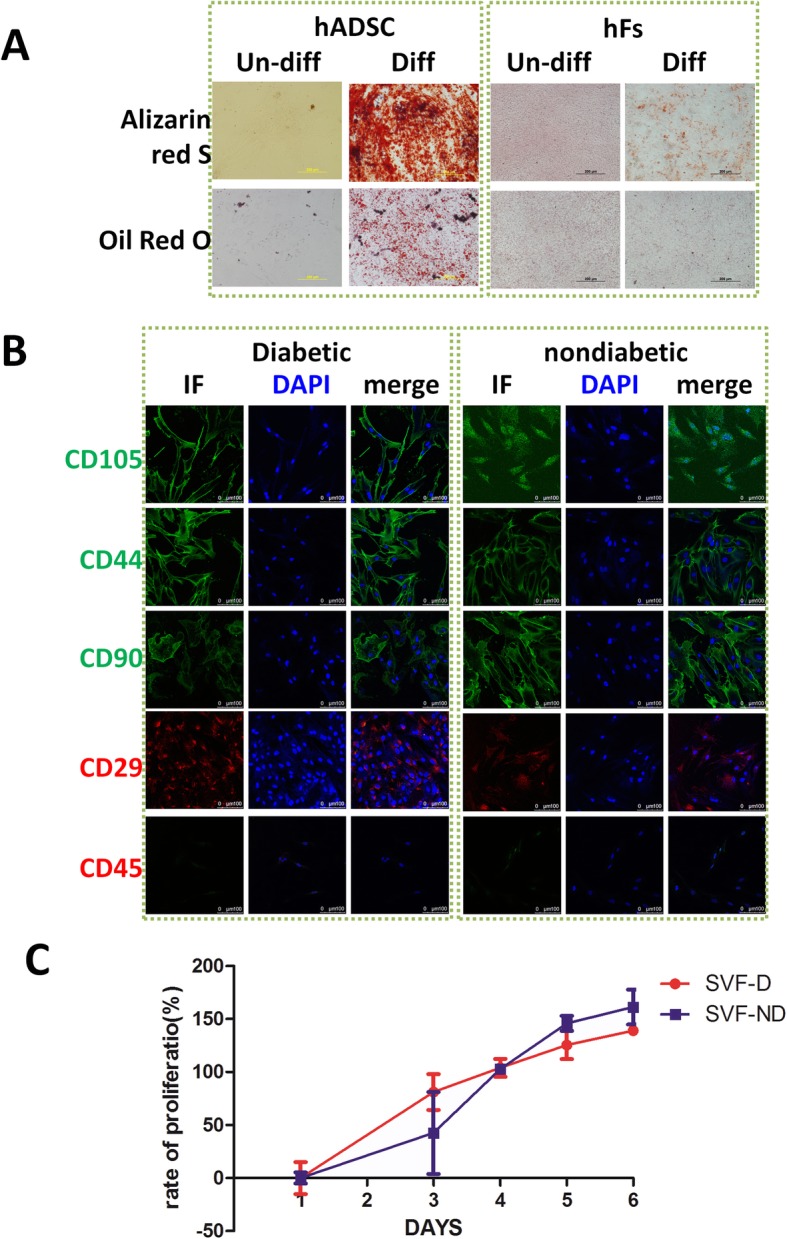
hADSCs have multiple characters of MSCs. **a** Osteogenic differentiation (upper panel) of hADSCs (left) and fibroblasts (hFs, right) stained with Alizarin red S and adipogenic differentiation (lower panel) of hADSCs (left) and fibroblasts (hFs, right) stained with Oil Red O. **b** Immunofluorescence staining of hADSCs from diabetic and nondiabetic donors for CD105 (green), CD44 (green), CD90 (green), CD29 (red), and CD45 (red) counterstained with DAPI (blue). **c** Cell proliferation of hADSCs measured by the MTS assay, with cell proliferation calculated as a percentage. The proliferative activity of hADSCs from diabetic patients (SVF-D) was not significantly different compared to those from healthy patients (SVF-ND)

The hallmarks of ADSCs are their characteristic surface protein expression, multipotency, and proliferative activity in vitro [23]. Freshly obtained SVF were immediately sorted and put into culture to observe their expansion, with adherent colonies forming after 24 h. Third passage hADSCs exhibited a fibroblast-like morphology. In order to evaluate the differentiation potential of hADSCs into adipocytes and osteoblasts, hADSCs were cultured in adipogenic or osteogenic inducing medium in vitro. Under lipid-forming conditions, hADSCs differentiate into adipocytes with Oil Red O (ORO)-positive lipid droplets. Osteogenic inducing medium promoted hADSCs to produce Alizarin red-positive particles. There changes were significantly different from those seen with hF cells (Fig. 3a).

The expression of surface proteins by hADSCs at passage 3 was examined using immunocytochemistry. Our cells fulfilled the defining criteria for MSCs according to the International Society for Cellular Therapy position statement. Our hADSCs are positive for CD105, CD44, CD90, and CD29 and have low expression of the negative marker CD45 (Fig. 3b). There was no significant difference between cells obtained from diabetic or healthy individuals regarding cell morphology or MSC marker expression.

To validate the proliferative capability of hADSCs, we tested the growth rate of hADSCs by MTS. Our results show that hADSCs have a good proliferative activity. The proliferative activity of hADSCs from diabetic patients (SVF-D) was not significantly different compared to those from healthy patients (SVF-ND). This result shows that hADSCs from diabetic patients did not exhibit an impaired proliferative activity (Fig. 3c). Taken together, these data suggest that hADSCs expanded from SVF of diabetic donors maintain MSC characteristics.

### Influence of SVF and hADSCs on wound healing processes in hyperglycemic mice

**Fig. 4:**
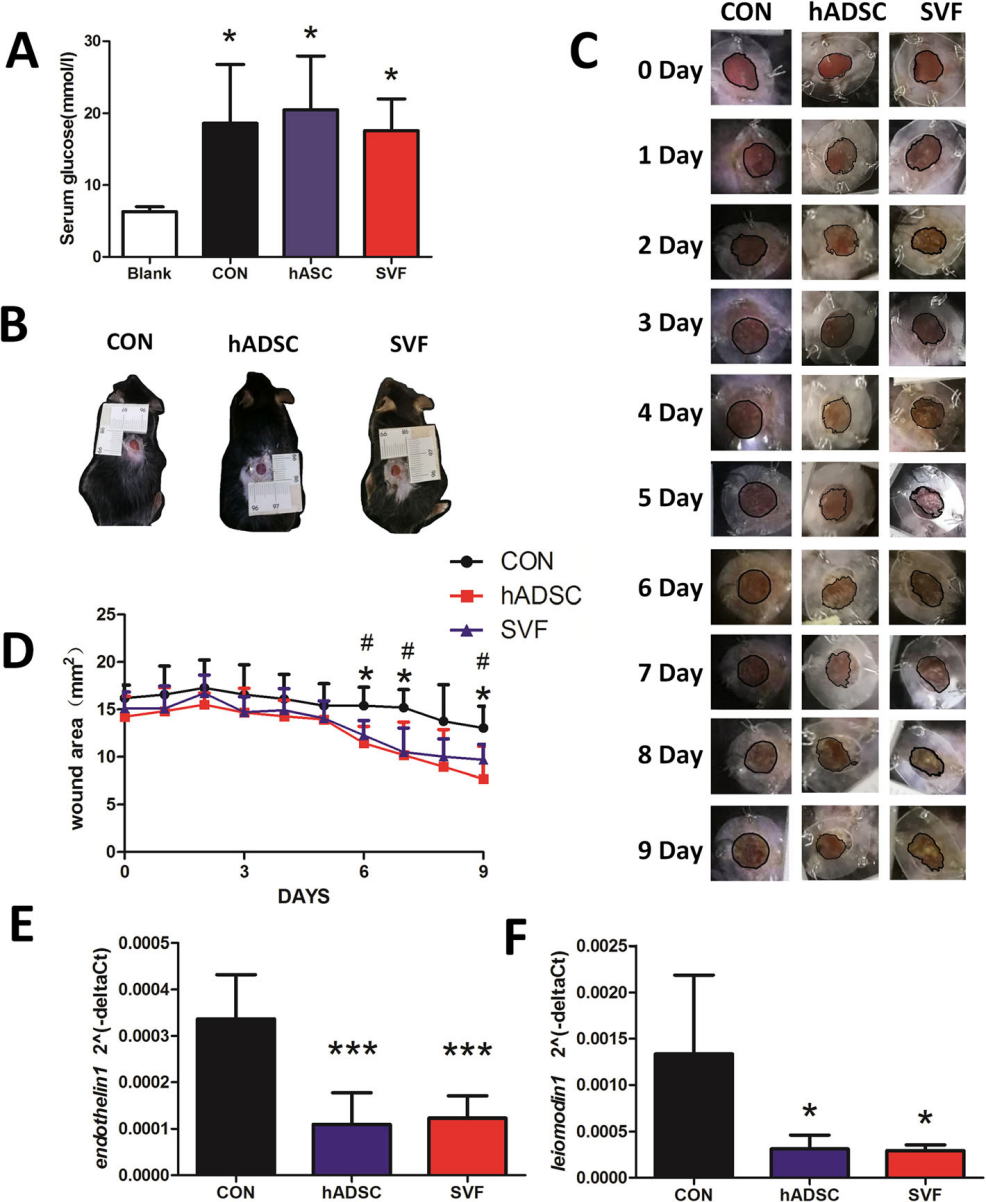
SVF and hADSCs improve the wound healing processes in hyperglycemic mice. **a** Fasted basal serum glucose levels treated with hADSCs and SVF (*P < 0.05, compared to blank). **b** Full-thickness excisional wounds of the same 6-mm-diameter size were created on the backs of each mouse. **c** Photographs of wounds 0–9 days after skin injury. **d** Quantification of the wound area, which was measured using the long and short diameters of the wounds, at different days after wounding. (*P < 0.05, hADSCs compared to control (CON); ^#^*P* < 0.05, SVF compared to CON). Influence of SVF and hADSCs on the expression of *Endothelin1* and *Leiomodin1* mRNAs. Gene expression of *Endothelin1* (**e**) and *Leiomodin1* (**f**) was evaluated by qRT-PCR from skin at the edges of the wounds treated or untreated with hADSCs or SVF. Results were normalized to *GAPDH* expression. (Statistical significance of the differences is indicated by ***P < 0.001, **P < 0.005, and *P < 0.05)

Hyperglycemic mice were induced by a single intraperitoneal injection of STZ. Serum glucose levels in these mice were significantly increased at 7 days after injection (Fig. 4a) and remained stable and high at 28 days. In this hyperglycemic mouse model, wound healing in the hADSC and SVF groups was enhanced compared with the control groups. As shown in Fig. 4c, the appearance of the wounds at different times post-wounding were quite different. Wound area at the different time intervals was determined. On days 6, 7, and 9 after wounding, wound area was significantly decreased after hADSC and SVF injection compared to untreated wounds at the corresponding times (Fig. 4d). Hence, hADSC and SVF cells significantly accelerated wound healing compared with the control group, especially at days 6, 7, and 9 post-surgery.

### Influence of SVF and hADSCs on mRNA expression in mouse skin

Expression of *Endothelin1* and *Leiomodin1* mRNAs in mouse skin were detected by qRT-PCR. *Endothelin1* and *Leiomodin1* mRNA levels were significantly decreased upon hADSC or SVF treatment (Fig. 4e, f).

### Influence of SVF and hADSCs on changes in skin histology

**Fig. 5:**
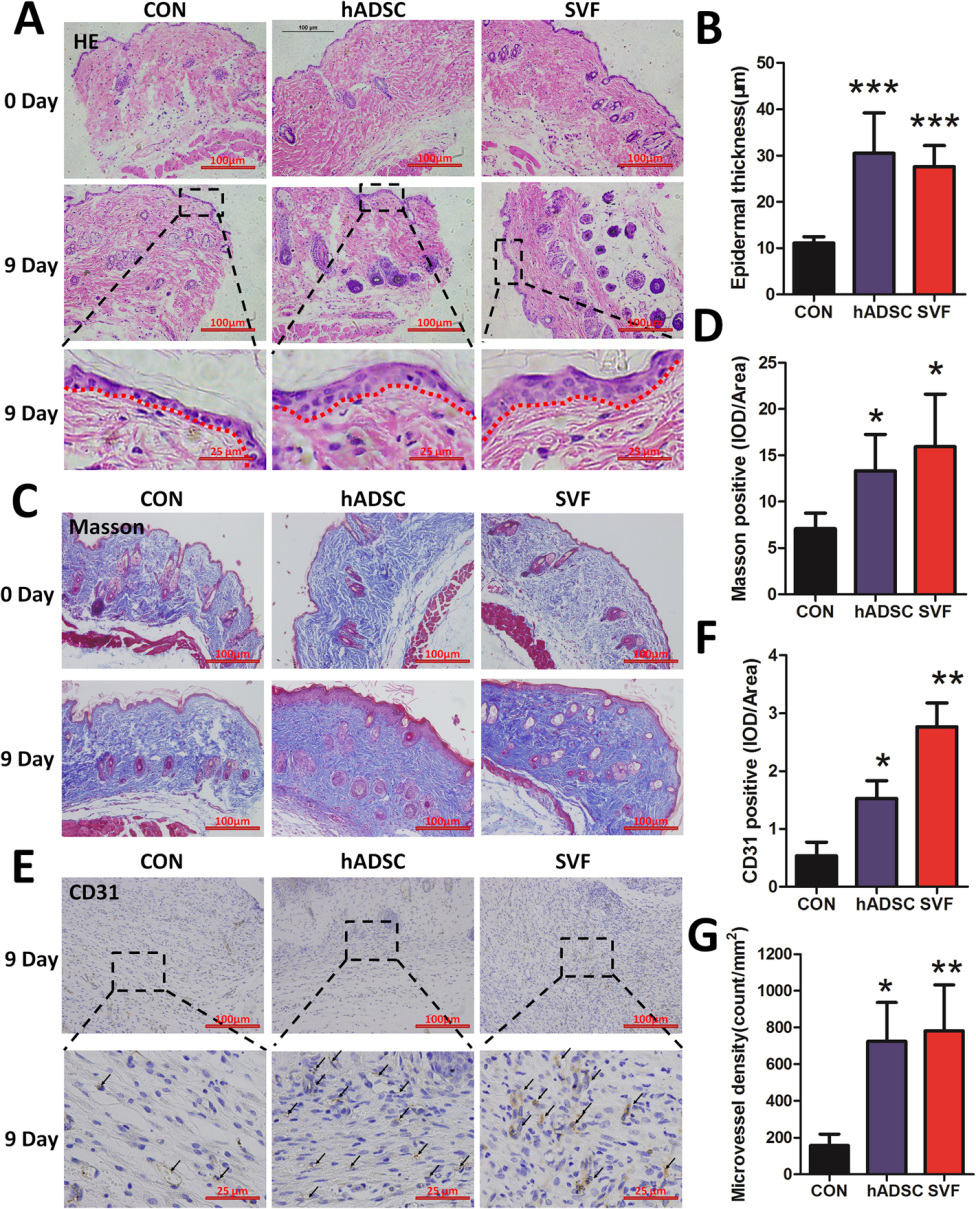
Histological changes induced in skin by hADSCs and SVF. **a** Hematoxylin and eosin (H&E) images of wound edge skin at days 0 and 9 post-injury. Dotted lines outline the boundary between the epidermis and the dermis. **b** Quantification of the epidermal thickness was performed using Image-Pro Plus 6. (***P < 0.001, compared to control (CON)). **c** Masson trichrome staining of sections of the wound edges from control and treated mice at 0 and 9 days after wounding. **d** Quantification of the Masson-positive area was performed using Image-Pro Plus 6. (*P < 0.05, **P < 0.005 compared to CON). **e** Immunohistological analysis of wound sections from control and treated mice 9 days after injury using antibodies directed against CD31. Arrows mark the CD31+ cells. **f** Quantification of the CD31-positive cells was performed using Image-Pro Plus 6 analysis software. (*P < 0.05, **P < 0.005 compared to CON). **g** Quantification of the microvessel density in sections as determined by CD31 expression (*P < 0.05, **P < 0.005 compared to CON)

We analyzed the histology of skin around the wounds 9 days after injury. When skin sections from the control (CON) were compared to the hADSC or SVF groups, we noted that in addition to the increased healing rate, the epidermis of the hADSC- or SVF-treated mice was thicker than that in controls (Fig. 5a, b). More collagen fibers were observed in the hADSC and SVF groups than in the control group on postoperative day 9, as detected by Masson staining (collagen appears blue). In the control group, disordered collagen and reduced levels of collagen in the skin around the wound were found (Fig. 5c). The content of collagen in the skin tissue was measured and compared with control mice; the collagen content of hADSC- or SVF-treated mice was significantly increased. These results indicate that hADSC or SVF treatment improves the collagen content of the skin, but no significant difference was seen between these two treatment groups (Fig. 5d).

To examine the effect of hADSCs and SVF on microvessel density, immunohistochemistry was performed to determine the levels of CD31 (Fig. 5e). Treatment with hADSCs or SVF significantly increased the number of CD31-positive microvessels in the dermis, numbers that were significantly higher than in the control group (Fig. 5f, g).

### Influence of SVF and hADSCs on differentially expressed genes in the skin of mice

**Fig. 6:**
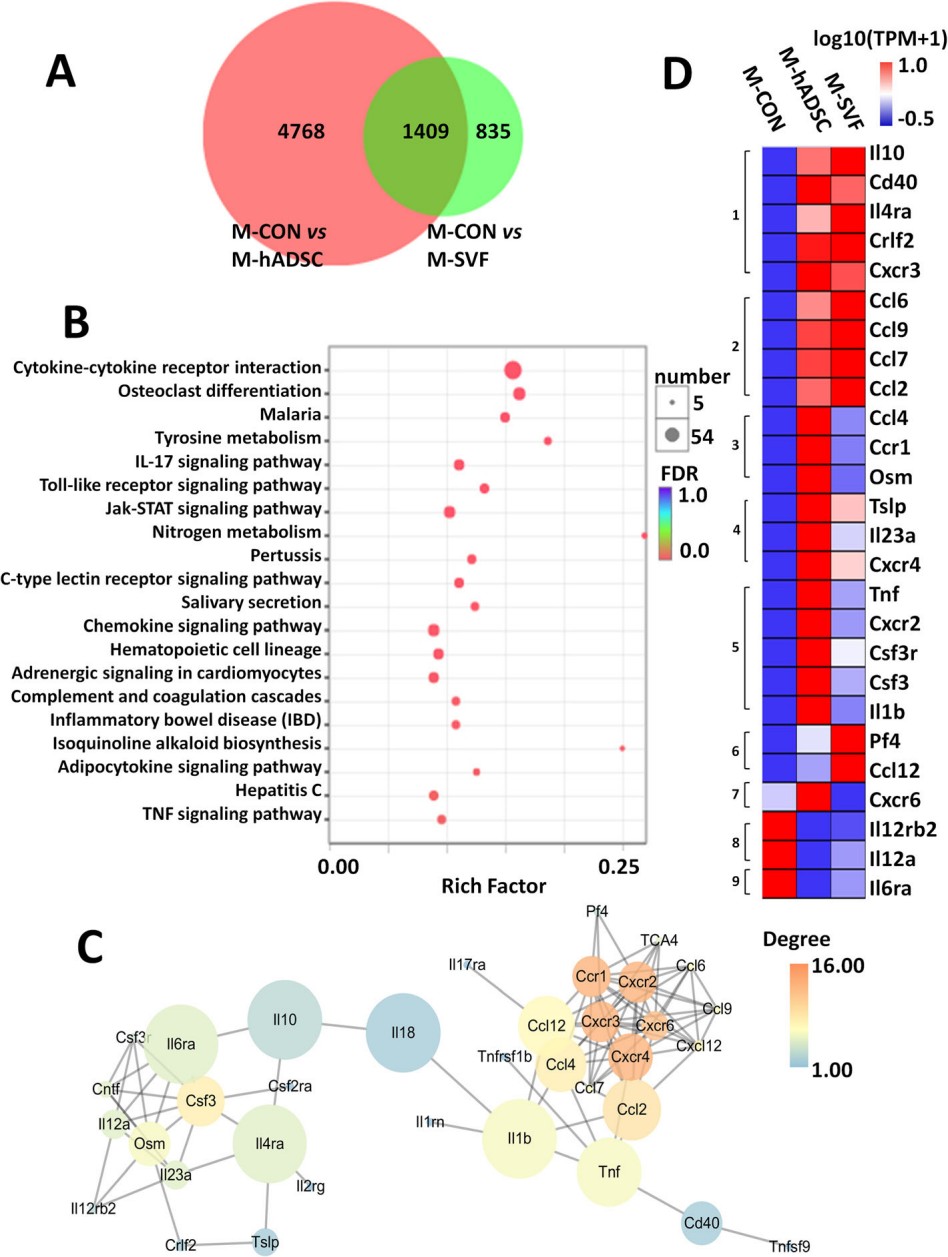
Analysis of differential gene expression in the skin from mice treated with hADSCs (M-hADSC) or SVF (M-SVF) compared to untreated skin (M-CON). **a** Venn diagram displaying the numbers and the overlap of the identified differentially expressed genes in M-hASC vs M-CON (red) and M-SVF vs M-CON (green). **b** Significantly enriched KEGG pathways. As shown in the bubble diagram, the *X*-axis represents the enrichment score of the gene sets, the *Y*-axis shows gene set names, and the size of the bubble indicates the number of genes in that gene set. KEGG pathways with *P* < 0.05 are significantly enriched. The top 20 significantly different KEGG terms are shown. **c** Expanded view of the network imported from Cytoscape, where nodes represent core differentially expressed genes in the gene set KEGG “cytokine-cytokine receptor interaction” pathway and edges the interaction. Node size represents “betweenness centrality,” node color represents “degree.” **d** Heat map of core differentially expressed genes in the gene set KEGG “cytokine-cytokine receptor interaction” pathway with Cytoscape network analysis. The colors in the heat map represent gene expression level (log10(TPM+1)). Red represents higher expression; blue represents lower expression. The brackets represent cluster analysis

To elucidate mechanisms involved in the improved wound healing due to hADSCs and SVF, we applied high-throughput RNAseq to investigate the gene expression profiles of skin after 9 days of treatment with hADSCs or SVF. Fold change was defined as the ratio of the mRNA level in hADSC or SVF group to the control group using FPKM. In this study, log2 (fold change) of > 1.00 and a *P* value < 0.05 were necessary to classify a gene as differentially expressed. A total of 6177 significantly differentially expressed genes were identified in the hADSC-treated mouse skin and 2244 in the SVF-treated mice, of which 1409 genes were significant in both the hADSC and the SVF groups, as shown in the Venn diagram in Fig. 6a.

The potential functions of genes that are differentially expressed in both hADSC and SVF groups were characterized based on their annotations in the KEGG database. Among the 325 enriched pathways, the 20 pathways with the higher enrichment indices are mainly associated with cytokine-cytokine receptor interaction, the IL-17 signaling pathway, the Toll-like receptor signaling pathway, and the Jak-STAT signaling pathway. A total of 54 genes matched to the “cytokine-cytokine receptor interaction” pathway, significantly more than for any other pathway. The largest spot (shown in red) in the KEGG enrichment diagram shown in Fig. 6b represents the “cytokine-cytokine receptor interaction.”

To analyze the functional significance of the changes in abundance of proteins due to the hADSC or SVF treatment, we used the STRING program to look for functional networks involved with the enriched genes in the KEGG gene set “cytokine-cytokine receptor interaction” pathway, which was visualized with Cytoscape. Figure 6c provides a graphical overview of the identified protein-protein interactions (PPI) and implicated changed behaviors in response to hADSC or SVF treatment due to these enriched proteins. The Cytoscape network analysis found that chemokines (including *ccl2*, *ccl4*, *ccl6*, *ccl7*, and *ccl9*), chemokine receptors (including *cxcr2*, *cxcr3*, *cxcr4*, *cxcr6*, and *ccr1*), interleukins (including *il1b*, *il12a*, and *il18*), and oncostatin M (*osm*) have high “betweenness centrality” and “degree” and play a key role in the PPI network (Fig. 6c).

Through a hierarchical clustering analysis (expression indicator: TPM; linkage rules: complete linkage; similarity measure: Euclidean), the 26 differentially expressed genes in the PPI network could be divided into 9 distinct clusters according to their expression profiles: 7 of which exhibited upregulated genes and 2 clusters exhibiting downregulated genes (Fig. 6d). This result is consistent with the observation that chemokines (including *Ccl2*, *Ccl4*, *Ccl6*, *Ccl7*, and *Ccl9*), chemokine receptors (including *Cxcr2*, *Cxcr3*, *Cxcr4*, *Cxcr6*, and *Ccr1*), interleukins (including *Il1b* and *Il18*), and oncostatin M (*Osm*) are upregulated and that *Il12a* is downregulated in mouse skin treated with SVF or hADSCs, compared to the untreated group (Fig. 6d). Among these genes, *Osm*, *Ccl4*, and *Ccr1* have the largest changes.

**Fig. 7:**
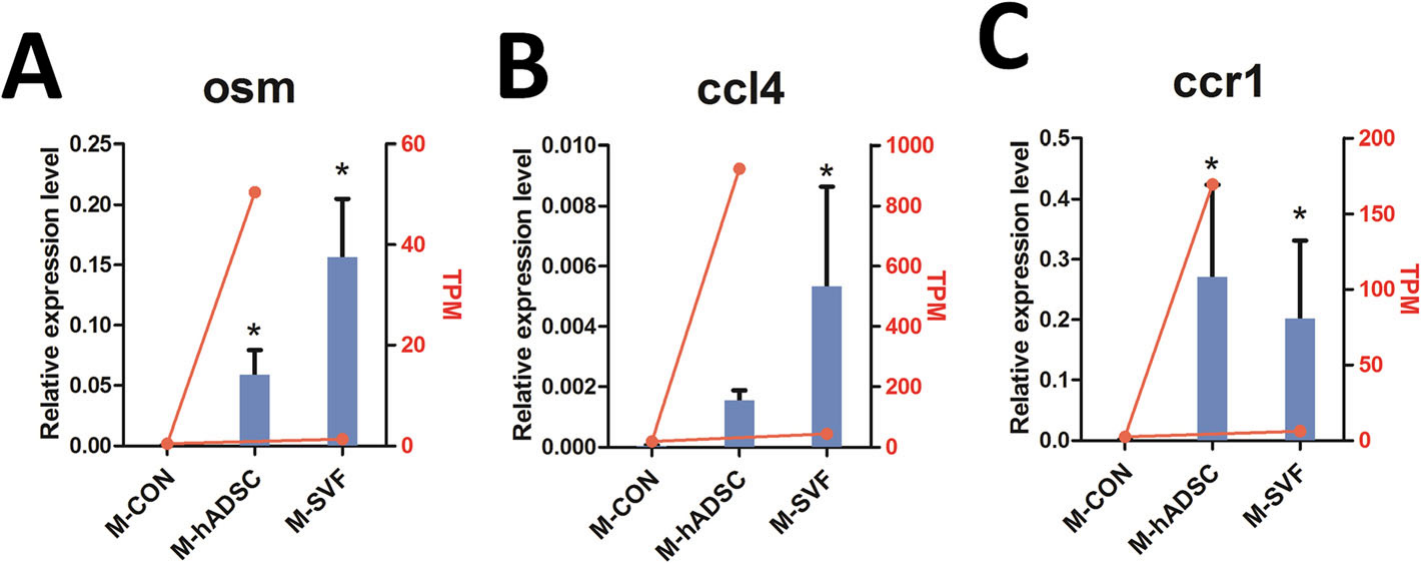
Comparison of the results obtained from RNAseq and qRT-PCR methodologies for three genes. Influence of hADSCs or SVF on *Osm* (**a**), *Ccl4* (**b**), and *Ccr1* (**c**) mRNA abundance. Relative gene expression evaluated by qRT-PCR is represented by the columns and TPM evaluated by RNAseq represented by the scatter diagram in skin treated or untreated with hASCs or SVF. qRT-PCR results were normalized to *GAPDH* expression. (*P < 0.05 compared to M-CON)

qRT-PCR was performed to verify the RNAseq results. When qRT-PCR was conducted, its results were consistent with the gene expression levels identified by RNAseq. In particular, qRT-PCR results showed that the gene expression levels of *Osm*, *Ccr1*, and *Ccl4* were significantly increased in skin from mice treated with hADSCs or SVF (Fig. 7).

### Influence of hADSCs on migratory ability of fibroblasts

**Fig. 8:**
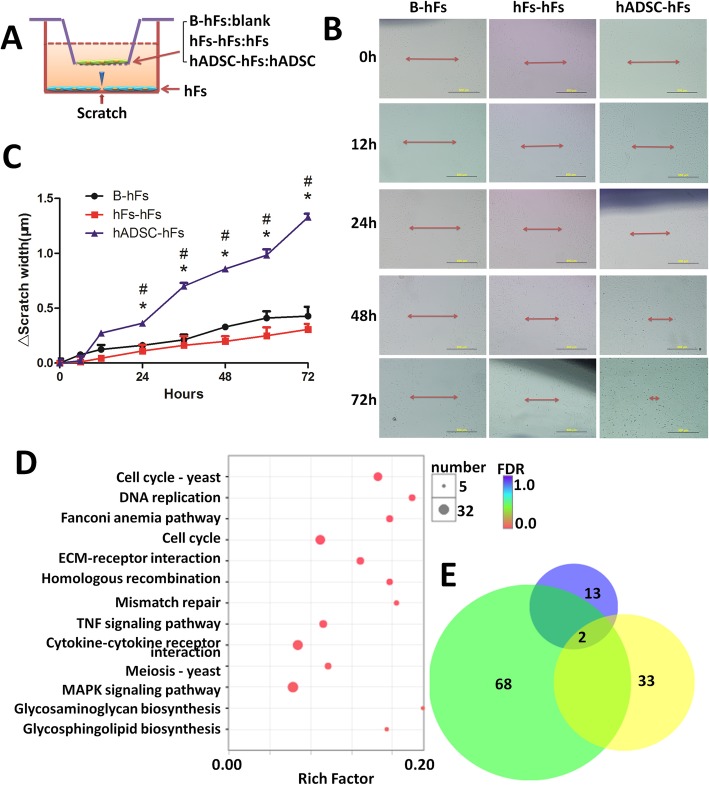
hADSCs promote migration of fibroblasts. **a** Diagram of the transwell setup. hF cells were seeded into the lower chamber of the 24-well transwell (pore size: 0.8 μm); hFs or hADSCs were plated in the upper chamber. **b** Representative images showing fibroblast scratches. Images were taken at 0 to 72 h. Arrow shows the width of the scratch wound. **c** Quantification of the scratch width over time measured using Image-Pro Plus 6 software. (*P < 0.05, compared to B-hFs; ^#^*P* < 0.05, compared to hFs-hFs). **d** Significantly enriched KEGG pathways are shown as a bubble diagram. The *X*-axis represents the enrichment score of the gene sets, the *Y*-axis shows gene set names, and the size of the bubble indicates the number of genes in that gene set. The KEGG pathways with *P* < 0.05 are significantly enriched. **e** Venn diagram displaying the number and overlap of the enriched KEGG pathway in the Fs-CON vs Fs-hADSC group (blue), M-CON vs M-hADSC group (green), and M-CON vs M-SVF group (yellow)

The scratch test and the transwell system (Fig. 8a) were used to investigate the influence of hADSCs on the migratory ability of fibroblasts. Results of the scratch test showed that the migratory ability of fibroblasts was significantly increased with hADSC co-culture at 24, 36, 48, 60, and 72 h compared to untreated controls (B-hFs and hFs-hFs). These data suggest that hADSCs functioned as pro-migration signals enhancing the migratory ability of fibroblasts. (Fig. 8b, c).

### Influence of hADSCs on the differential expression profile of fibroblasts

To clarify mechanisms involved in the migration promotion of hADSCs, we used high-throughput RNAseq and calculated the relative expression levels of genes with FPKM to study the effect of 72 h of hADSC treatment on gene expression in fibroblasts. In this study, log2 (fold change) of > 1.00 and a *P* value < 0.05 were required to classify differentially expressed genes. A total of 1720 unique differentially expressed genes in fibroblast cells treated with hADSCs were identified.

The annotations of genes that were differentially expressed between the hADSC-treated and control cells were obtained from the KEGG database. Significantly enriched pathways (corrected *P* value < 0.05) among the 326 successfully mapped pathways are mainly associated with the cell cycle–yeast, DNA replication, cell cycle, extracellular matrix (ECM)-receptor interaction, homologous recombination, mismatch repair, TNF signaling pathway, cytokine-cytokine receptor interaction, and MAPK signaling pathway (Fig. 8d).

A requirement that a KEGG pathway be regulated by all of treatment groups, including mice wound skin with hADSCs and SVF, and fibroblasts treated with hADSCs, increases the likelihood that the identified KEGG pathway is consistently affected in the fibroblasts both the in vivo and the in vitro models. Using this strategy, 68 KEGG pathways were found to be significantly enriched in the hADSC-treated mice skin, 33 pathways in the SVF-treated mice skin, and 13 pathways in the hADSC-treated fibroblasts cells, with 2 of these pathways overlapping in all of these groups. The two pathways were ECM-receptor interaction (Pathway ID: map 04512) and cytokine-cytokine receptor interaction (Pathway ID: map 04060). We generated Venn diagrams to show the extent of similar response in the in vivo and in vitro models (Fig. 8e). There are 16 differentially expressed genes matched to the “ECM-receptor interaction” pathway and 29 genes matched to the “cytokine-cytokine receptor interaction” pathway in the fibroblast cells.

### Influence of hADSCs on HUVEC capillary structure formation

**Fig. 9:**
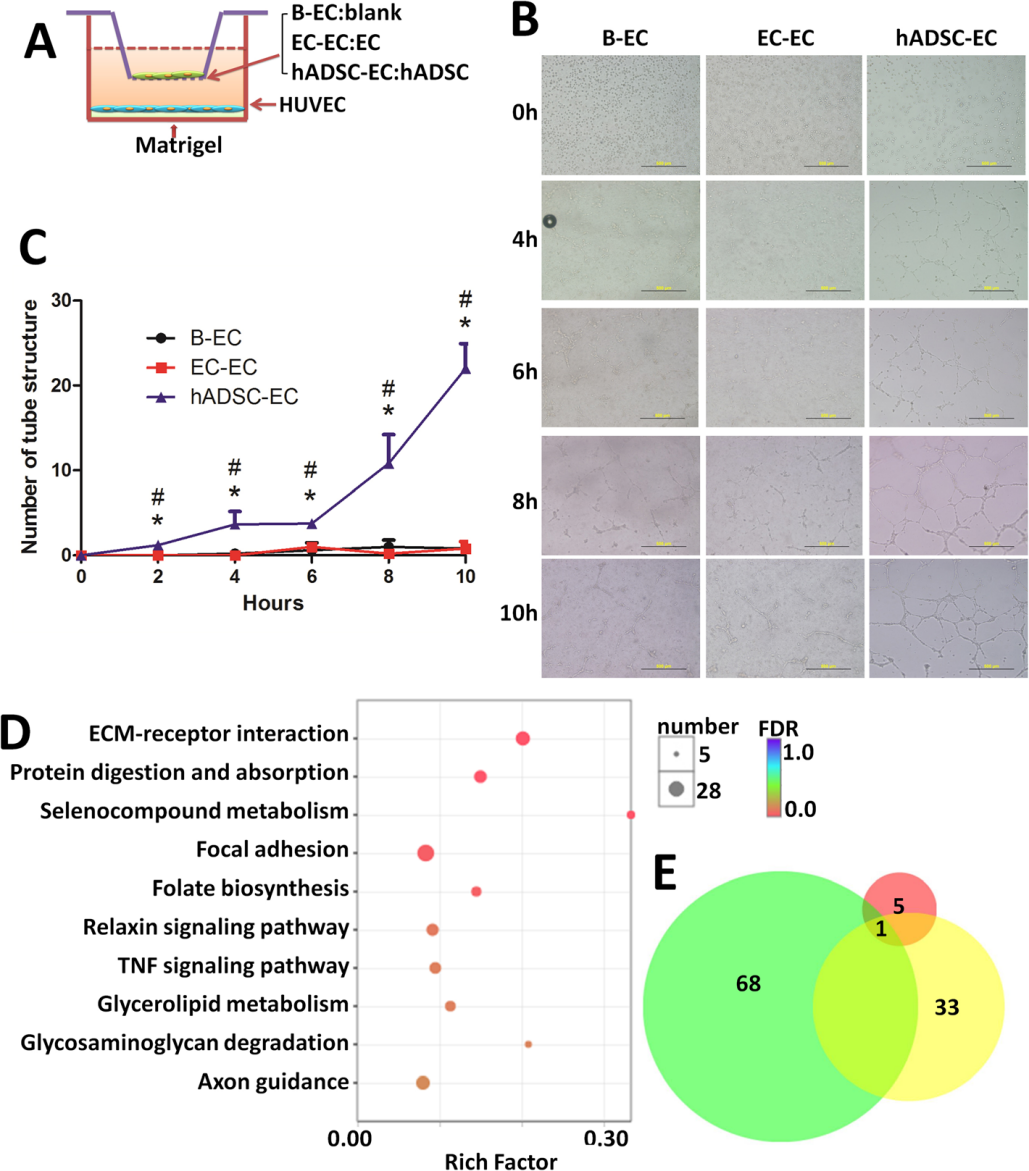
hADSCs promote tube formation. **a** Diagram of the transwell setup. HUVECs were seeded into the lower chamber of the transwell (pore size: 0.8 μm) with HUVECs or hADSCs plated in the upper chamber. **b** Representative images showing endothelial cell tube formation. Images were taken at 0 to 10 h. **c** Numbers of tube structure over time were counted. (*P < 0.05, compared to B-EC; ^#^*P* < 0.05, compared to EC-EC). **d** Significantly enriched KEGG pathways are shown as a bubble diagram. The *X*-axis represents the enrichment score of the gene sets, the *Y*-axis shows gene set names, and the size of the bubble indicates the number of genes in that gene set. The enrichment score was calculated using the total number of genes in each functionality group and the total number of genes that could be functionally annotated. *P* < 0.05 was used as the thresholds in selecting significant KEGG pathways. The significantly different KEGG terms are shown. **e** Venn diagram displaying the number and overlap of the enriched KEGG pathway in EC-CON vs EC-hADSC group (red), M-CON vs M-hADSC group (green), and M-CON vs M-SVF group (yellow)

During wound healing, new blood vessels are needed to ensure sufficient blood flow to supply oxygen and nutrients for the metabolism of the various types of cells around the wound. An important process in angiogenesis is the formation of tubule structures by endothelial cells accompanied by the migration of fibroblasts [24]. To characterize the angiogenesis process, we studied the promotion of endothelial cell tubulegenes by hADSCs (Fig. 9a). We found that hADSCs promoted the formation of tubules after 2 to 10 h of treatment compared to controls (B-EC and EC-EC) (Fig. 9b). Counting of the number of branches in the tubes generated in the co-culture with hADSCs revealed that the hADSC co-culture significantly increased the number of new tube-like structures compared to those generated by untreated HUVECs (Fig. 9c).

### Influence of hADSCs on the differential expression profile of endotheliocyte

To elucidate the pro-angiogenic mechanisms of hADSCs, we applied high-throughput RNAseq to investigate the effect of hADSC treatment for 72 h on EC gene expression. In this study, log2 (fold change) of > 1.00 and a *P* value < 0.05 were necessary to classify differentially expressed genes. A total of 1577 genes were identified in EC treated with hADSCs, which were then used for GO, KEGG, and PPI analyses.

Genes that were differentially expressed between the hADSC and control treatments were annotated using the KEGG database. Significantly enriched pathways (corrected *P* value < 0.05) among the 327 successfully mapped pathways are mainly associated with the ECM-receptor interaction, protein digestion and absorption, selenocompound metabolism, focal adhesion, folate biosynthesis, relaxin signaling pathway, and TNF signaling pathway (Fig. 9d).

Only one KEGG pathway affected in EC overlapped with both in the in vivo and in vitro models, including mice wound skin with hADSCs and SVF and EC treated with hADSCs. The overlapping KEGG pathway was ECM-receptor interaction (Pathway ID: map 04512). We drew Venn diagrams to show the extent of similarities of the responses of the in vivo and in vitro models (Fig. 9e). A total of 20 differentially expressed genes matched to the “ECM-receptor interaction” pathway in endotheliocyte.

### Influence of hADSCs on the “ECM-receptor interaction” pathway

**Fig. 10:**
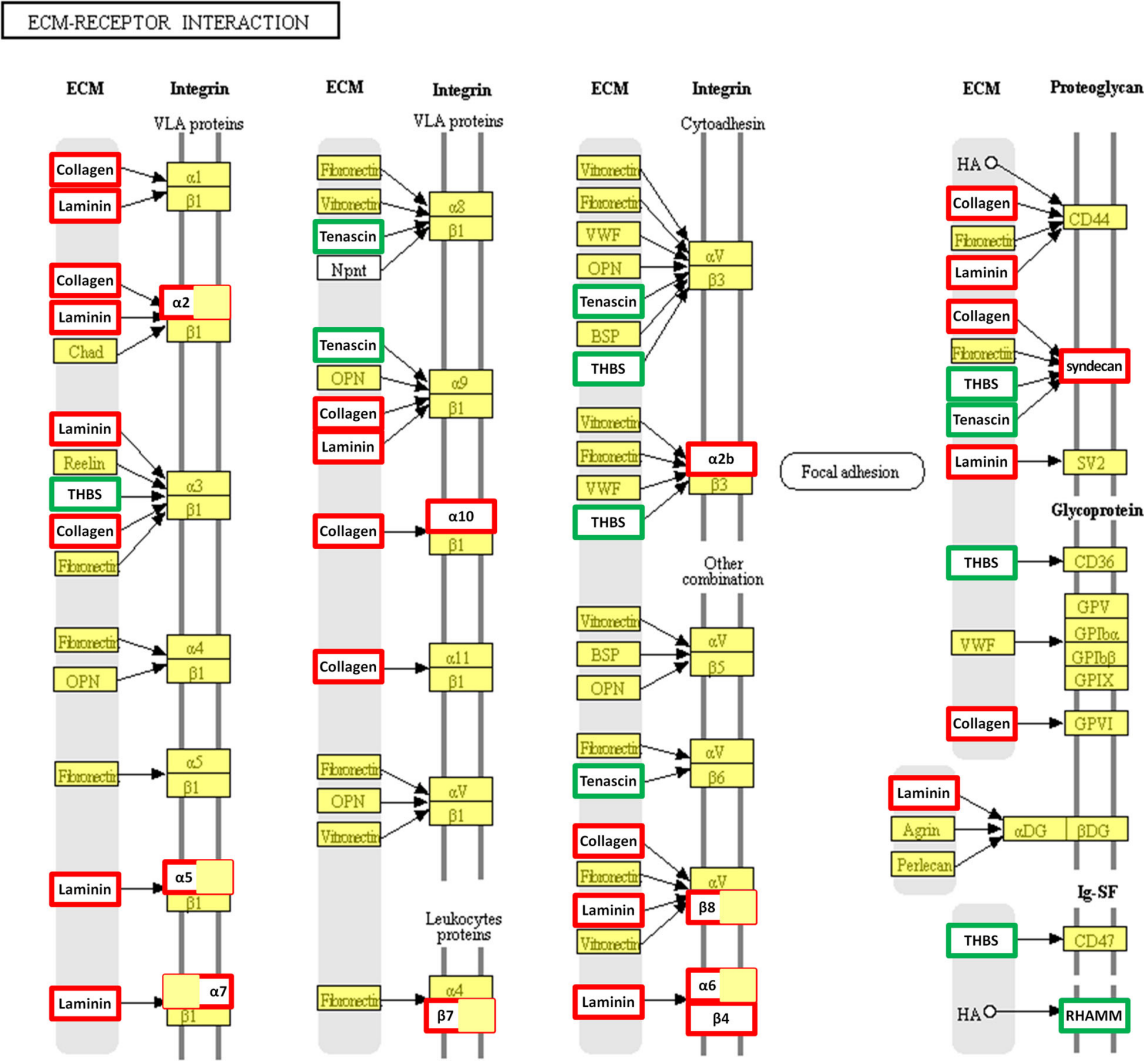
Differentially expressed genes in the ECM-receptor interaction pathway are shown. The ECM-receptor interaction pathway is adapted from the KEGG database. Genes upregulated in endotheliocytes and fibroblast by hADSCs are in (red), only upregulated in fibroblast are in red with right half filled with yellow, only upregulated in endotheliocytes are in red with left half filled with yellow, and downregulated are in green. Gene with log2 (fold change) > 1.00 is considered to be a significantly differentially expressed gene

Our KEGG analysis showed that the, in vivo and in vitro, hADSC-treated fibroblasts and endotheliocyte all had similar genetic changes in the KEGG pathway “ECM-receptor interaction.” In the current study, we verified that the ECM-receptor interaction-related genes, including *integrins* (*Itga7*, *Itga2b*, and *Itgb4*), *laminin* (*Lamb3* and *Lamc3*), and *collagen* (*Col6a3*, *Col1a1*, *Col9a3*, and *Col4a2*), had expression levels that were significantly upregulated and that *thrombospondins* (*Thbs1* and *Thbs3*) were downregulated in fibroblasts and endotheliocyte treated with hADSCs. The location and role of these different genes of fibroblasts and endotheliocyte in the KEGG pathways were analyzed (Fig. 10), and it was found that these genes play an important role in extracellular matrix function.

**Fig. 11:**
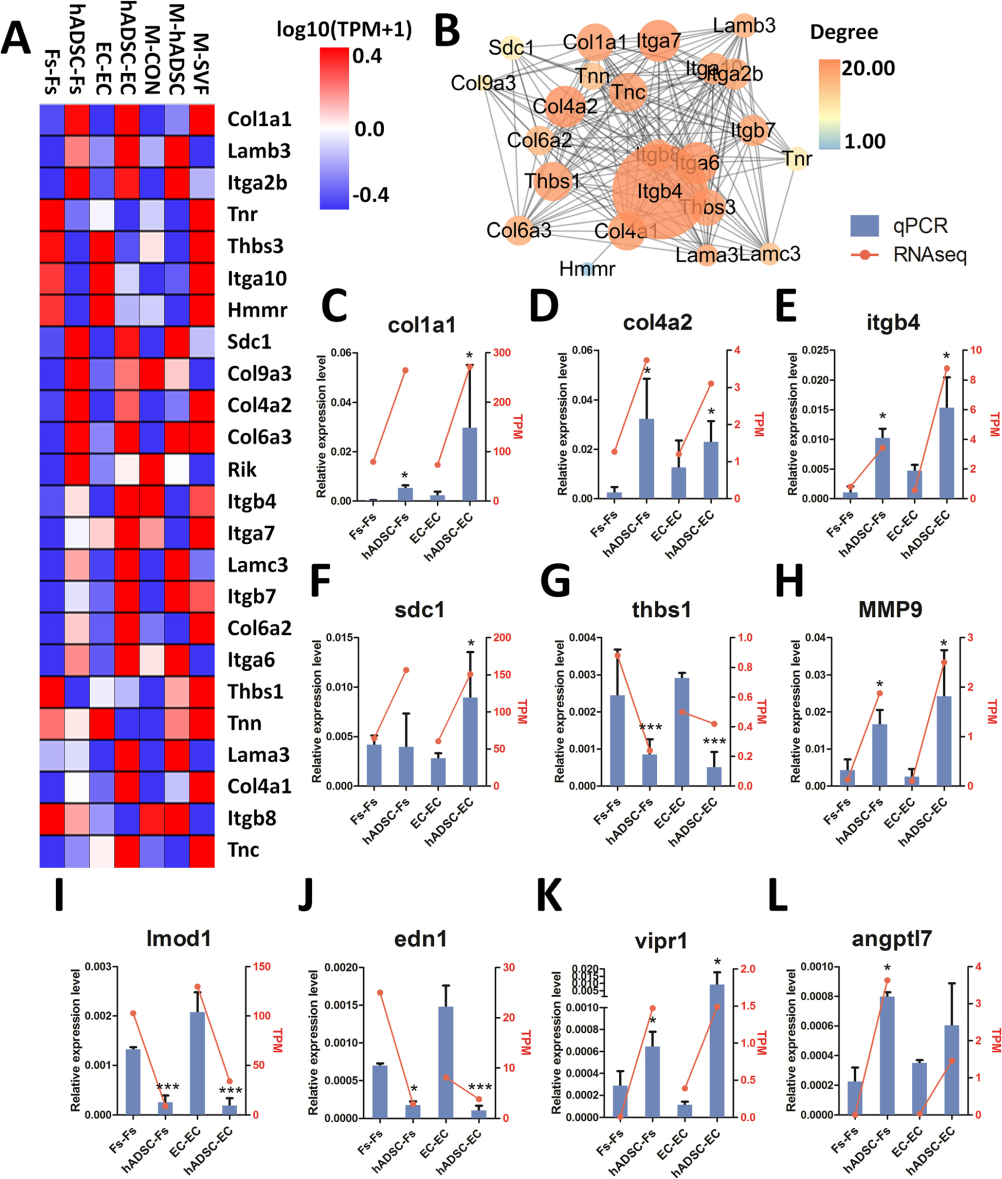
hADSC-related gene-regulatory network in “ECM-receptor interaction.” **a** Heat map of core differentially expressed genes in the gene set KEGG “ECM-receptor interaction” pathway. The colors in the heat map represent gene expression level (log10(TPM+1)). Red represents higher expression, blue represents lower expression. **b** Expanded view of the network imported from Cytoscape, where nodes represent core difference gene in the gene set KEGG “ECM-receptor interaction” pathway and edges the interaction. Node size represents “betweenness centrality,” node color represents “degree.” **c**–**l** Comparison of the results of 10 genes obtained using RNAseq and qRT-PCR methodology. Relative gene expression was evaluated by qPCR in Fs and EC treated or untreated with hADSCs. Results were normalized to GAPDH expression (***P < 0.001, **P < 0.005, *P < 0.05)

To explore the functional consequences of the changes in gene expression associated with hADSCs, we analyzed the enrichment genes in the gene set in the KEGG pathway “ECM-receptor interaction” (Fig. 11a). We used the STRING programs to look for functional networks of these enriched genes, which was visualized with Cytoscape. Figure 11b provides a graphical overview of the STRING results and indicates that the changed behavior in response to hADSC treatment in proteins. The Cytoscape network analysis found that genes, including *Itgb4*, *Itga7*, *Itga6*, *Col4a2*, *Thbs1*, *Tnc*, *Col4a1*, *Col1a1*, *Thbs3*, and *Itgb7*, have a high “betweenness centrality” in the PPI networks of “ECM-receptor interaction.”

### Influence of hADSCs on gene expression in fibroblasts and endotheliocytes

qRT-PCR was performed to verify the RNAseq results and was found to be consistent with the RNAseq results. Genes to be confirmed were selected based on their importance and likely functions for wound healing. qRT-PCR results show that the expression levels of genes associated with extracellular matrix, including *Col1a1*, *Col4a2*, *Itgb4*, *Sdc1*, and *Mmp9*, were significantly increased in both fibroblasts and endotheliocyte treated with hADSCs. Meanwhile, *Thbs1* was significantly decreased in fibroblasts and endotheliocyte treated with hADSCs. In addition to this, *Lmod1 (Leiomodin1)* and *Edn1 (Endothelin1)* were significantly decreased in fibroblasts and endotheliocyte treated with hADSCs. In contrast, gene expression levels of *Vipr1 (Vasoactive intestinal polypeptide receptor 1)* and *Angptl7* (*Angiopoietin-like 7*) were significantly increased in the fibroblasts and endotheliocyte treated with hADSCs. The results of qRT-PCR are shown in Fig. 11c–l, and the expression patterns of these 10 genes are consistent with the RNAseq results.

## Discussion

The stromal vascular fraction (SVF) obtained by collagenase digestion and centrifugal separation contains a variety of biologically active cells [25]. A subgroup of SVF cells have a large proportion of characteristics similar to those of mesenchymal stem cells from bone marrow sources, and are named “adipose-derived stem cells (ADSCs)” [26]. They are an ideal source of stem cells [25]. SVFs collected according to a normal operation method can promote the survival of transplanted fat and assist in the treatment of refractory wound. In animal tests, SVF was found to promote angiogenesis, increase vascular density, and improve blood supply to ischemic myocardium. It is well known that the purity and number of stem cells obtained by the direct separation method are relatively low, and it takes a long time to purify and cultivate them in vitro. During this process, it is possible that these cells become infected by microorganisms such as bacteria. These factors weaken the potential of ADSC-based cell therapy. In contrast to ADSCs, SVF can be isolated in real time and contains a sufficient number of cells to eliminate the need for in vitro amplification. Therefore, SVF could be an ideal cell source for locally applied treatment of refractory wounds [27].

### hADSCs have multiple characteristics of MSCs

Our results show that hADSCs expanded to the third passage have characteristics similar to those of the MSCs. Immunocytochemical tests show that hADSCs express high levels of CD105, CD44, CD90, and CD29 and are CD45 negative. Additionally, hADSCs expanded from SVF in vitro had adipocytic and osteoblastic differentiation potential. In vitro, amplified hADSCs also have a vigorous proliferation potential. The proliferative activity of hADSCs from different patients was significantly different; however, hADSCs from a diabetic patient did not exhibit an impaired proliferative activity. This provides an experimental basis for patients with diabetes to carry out autologous SVF transplantation for wound healing. In addition to multipotency, an expanded ADSC exerts paracrine effects to regulate the function and mRNA expression of fibroblasts and endothelial cells.

### hADSCs and SVF significantly improve the wound healing processes by regulating the “cytokine-cytokine receptor interaction” pathway

In this study, using a mouse model of skin wound healing, we investigated the role of hADSCs and SVF in regulating the wound healing processes and its mechanisms. SVF or hADSCs were subcutaneously injected to determine their effects on skin wound healing, allowing observation of changes to skin structure after healing. Our results show that wound healing in SVF-treated mice was significantly more rapid than in control mice, but with no significant difference from the hADSC-treated group. Similar to the literature [28], our results show that SVF contains a percentage of hADSCs. Considering that SVF and hADSC groups use the same number of cells, therefore, it can be inferred that hADSCs in SVF have better activity than expanded hADSCs. An increase in epidermal thickness, compared to control, was found with SVF or hADSC treatment. Skin contains many types of cells, including dermal fibroblasts that participate in the synthesis of extracellular matrices, secrete growth factors, and play an important role in wound healing [29]. The thickness of skin is likely associated with the proliferation of fibroblasts and other skin cells stimulated by SVF. Angiogenesis and the migration of fibroblasts are essential in wound healing by allowing an increase in the blood supply near the wound to improve cell proliferation and metabolism. Therefore, improving the function of fibroblasts and endotheliocytes and promoting angiogenesis are of great significance to wound healing [30]. Our Masson stains and CD31 immunohistochemistry experiments showed that hADSCs and SVF significantly induced collagen levels and microvascular formation in skin wounds, which should strongly promote wound healing. Taken together, hADSCs and SVF promote wound healing by inducing the angiogenic process and collagen production.

Leiomodin1 is an important factor in the contractile activity of smooth muscle cells and maintains vascular homeostasis [31]. Leiomodin has three isoforms, with Leiomodin1 being mainly expressed in the smooth muscle cells found in vascular tissue. Although the structure, function, and distribution of the different leiomodin isoforms vary greatly between tissues, experiments have shown that actin polymerization is strongly promoted by Leiomodin in vitro [32]. Actin polymerization can regulate smooth muscle tension and induce vasoconstriction [33]. Endothelin1 is a strong vasoconstrictor that is secreted by vascular endothelial cells, which is a powerful vasoconstrictor in mammals. A high level of endothelin1 results in vasoconstriction and decreased compliance of the blood vessel walls and is associated with inflammation and thrombosis, which can lead to heart failure, hypertension, and atherosclerosis [34]. Our data revealed that treatment with hADSCs or SVF decreased the expression of *Endothelin1* and *Leiomodin1* mRNAs, which potentially improves blood supply to the wound by inhibiting the contraction of local blood vessels.

RNAseq and network analysis of gene expression profiles of skin tissue around the wound found that the expression of 54 genes in the “cytokine-cytokine receptor interaction” pathway has changed after SVF or hADSC treatment for 9 days. Chemokines play an important role in regulating multiple processes in wound healing [35], which is consistent with the observation that *chemokines*, *chemokines receptor*, *interleukins*, and *oncostatin M* (*Osm*) were upregulated in mouse skin treated with SVF or hADSCs. Among these genes, expression levels of *Osm*, *Ccl4*, and *Ccr1*, which have the largest changes, were analyzed by qRT-PCR, which was consistent with the RNAseq results.

Various types of inflammatory cells and factors are involved in inflammatory disorders, which contribute to delayed wound healing. OSM, an Il-6 family protein, is secreted by activated macrophages and inhibits the expression of inflammatory cytokines TNFα and Il-1β at wound sites. In the late stages of inflammation, OSM changes the behavior of the inflammatory factors and induces an anti-inflammatory reaction, thereby promoting wound healing [36]. Small molecular weight chemotaxic cytokines are called chemokines. In injury, a variety of cells, including endothelial cells and fibroblasts, secrete cytokines with molecular weights between 8 and 12 kDa [30]. Chemokines can promote angiogenesis and immunoregulation in all stages of wound healing, with several contributing to multiple stages of wound healing. CCL4, also referred to as MIP-1β (macrophage inflammatory protein-1β), is a major chemokinetic factor associated with the recruitment of monocytes. Chemokine receptor 1 (CCR1), expressed on the surface of mononuclear cells, interacts with its ligands CCL3 and CCL5 [37]. Once monocytes enter a wound, they differentiate into macrophages, secrete growth factors, and function as antigen-presenting cells and scavenger cells. A study by Kaesler et al. showed that chemokine CCL-6 is a powerful chemokinetic factor secreted by macrophages. CCL6 expression increases during skin injury and through chemotaxis attracts a large number of macrophages, which increases the number of macrophages in the healing of skin wounds [37]. A variety of CXCs promote the regeneration of blood vessels around the wound with studies showing that local levels of CXCL1, CXCL2, CXCL5, CXCL7, CXCL8, and CXCL12 increase during wound healing [30].

In our results, CC-chemokines were upregulated in wound healing, including CCL2, CCL4, CCL6, CCL7, and CCL9, which are all able to chemoattract macrophages; simultaneously, OSM, produced by activated macrophages, is also upregulated. This suggests a high content of macrophages in the wound. These data demonstrate that macrophage chemotaxis by SVF or hADSCs can offer a potential therapeutic benefit for wound healing.

### hADSCs improve angiogenesis by regulating the “ECM-receptor interaction” pathway

New blood vessels help transport nutrients and oxygen to the wound area. In the wound healing process, growth of microvessels of the size of capillaries is required [38]. The proliferation and subsequent apoptosis of endotheliocyte, the proliferation and migration of fibroblasts, and the synthesis of collagen are all required to simultaneously occur in a coordinated fashion to promote wound closure. The timing of the changes in the levels of a variety of soluble factors, such as VEGF and chemical chemokines (CXCL10, CXCR3 ligands), are carefully regulated to induce the growth of new blood vessels in the early stages of injury and their degradation in the late stages of healing [39]. These factors are also associated with angiogenesis as well as the chemotaxis and activation of macrophages. Our study found that 9 days after injury, the expression of multiple chemokines and the density of new capillaries in the wound locally increased. Vascular and non-vascular cells coordinated to promote physiological healing of wounds. Interaction between capillary endothelial cells and peripheral cells are required to determine the remodeling process of microvessels [38]. Evidence suggests that microvascular dysfunction is responsible for the pathogenesis of diabetic wound healing [3]. The function of endothelial cells, the basic component of blood vessels, is an important factor affecting angiogenesis. We found that HUVECs treated with hADSCs increased in tubulogenesis ability.

The connective tissue contains a large number of fibroblasts, accounting for a majority of their cellular components. By synthesizing ECM in the dermis, fibroblasts maintain the normal structure of ECM in normal and injured tissues, thereby promoting wound healing [40]. Fibroblasts accelerate the damage repair process by increasing matrix synthesis, secreting growth factors, and promoting wound contraction. We found that fibroblasts treated with hADSCs have increased migratory ability. In the process of angiogenesis, fibroblasts and vascular endothelial cells bond, and chemovascular signals, including growth factors, activate the migration of vascular endothelial cells, which eventually forms a comprehensive vascular network [41]. ECM plays an important role in providing a place for cell adhesion and participates in the formation of new blood vessels [42].

We used RNAseq and network analysis tools to study pathways affected by hADSCs. KEGG analysis found that the hADSC-treated fibroblasts and endotheliocytes had similar genetic changes in the “ECM-receptor interaction” pathway. By directly interacting with cell surface receptors, ECM provides a substrate for cell adhesion and migration and participates in the repair or regeneration of damaged skin tissue [43]. Recent studies have found that some ECM proteins regulate the distribution and bioavailability of growth factors, promoting wound healing through their involvement in controlling the core behaviors of cells [44]. In our study, we verified that ECM-receptor interaction-related genes, including *collagen*, *laminin*, *integrins*, and *syndecans*, have significantly upregulated expression levels and that *thrombospondins* were downregulated. *Itgb4*, *Itga7*, *Itga6*, *Col4a2*, *Thbs1*, *Tnc*, *Col4a1*, *Col1a1*, and *Thbs3* play key roles in the PPI networks identified in fibroblasts and endotheliocytes after treatment with hADSCs. These regulated genes participate in cellular pathways closely associated with wound healing.

Collagen consists of type I collagen (about 80%) and type III collagen (about 10%), which forms a crosslinked network. These two types of collagen are the main fibrins making up healthy dermal ECM [45]. In this study, *type I collagen alpha 1* (*Col1a1*) was significantly upregulated not only in mice with skin wounds, but also in fibroblasts and endotheliocytes treated with hADSCs and SVF. *Col4a2* and *3*, *Col6a2* and *3*, and *Col9a3* were also upregulated by hADSCs in fibroblasts and endotheliocytes. The fibrin molecule surrounds the periphery of the elastic fiber bundle composed of highly crosslinked elastic protein cores and constitutes a bundle of elastic fibers. These elastic fibers are mixed with collagen networks to form fibers that are necessary for skin extension and compliance. After skin injury, the extracellular matrix in dermis is destroyed with the formation of new fibrin matrix [45]. Importantly, fibrin and other proteins in the collagen-elastic protein networks interact with soluble signaling molecules (such as growth factors) and present cell adhesion sites [45]. The most abundant glycoprotein in the basal membrane ECM is laminins. Laminins are cruciform trimers whose surface is a three-stranded spiral structure that is widespread in a variety of organizations. The laminin structure consists of three chains, α, β, and γ, and is accordingly named by its αβγ chain. Cellular receptors for laminins include the integrins and syndecans. Laminins are involved in regulating core cellular activity, such as cell adhesion and migration, cell apoptosis, proliferation, and differentiation, and provide a structural scaffold for cells. As such, in skin wound healing, laminins play a critical role in re-epithelialization and angiogenesis. Studies have shown that the rate of protease hydrolysis of LAMA3 is related to wound healing. Laminin has a high affinity with a variety of growth factors, and the α chain laminin type G domain is the binding domain with heparin. These regions promote the attachment of endothelial and fibroblasts cells by binding to the cell surface receptor syndecan. The application of laminin for wound healing in type 2 diabetic mice can significantly increase the peripheral growth factor concentration and promote wound healing [44]. Here, we showed that *Lama3*, *Lamb3*, and *Lamc3* were significantly upregulated in fibroblasts, endotheliocytes, and mice wound skin after treatment with hADSCs or SVF. This upregulation of laminin may be clinically useful and lead to effective tissue regeneration.

Integrin is the main type of transmembrane cell surface receptor involved in the interaction between cells and the extracellular matrix. Collagen and glycoprotein in the ECM have multiple cell-binding sites, including those for integrin receptors, and provide platforms for migrating cells [30]. Integrin can regulate cell adhesion and migration as well as other cellular processes, including, for example, proliferation and differentiation [46], by identifying short sequences present in the extracellular matrix, including collagen, histone, and fibrin [30]. In the current study, we showed that five members of integrin family, including *a2b*, *a6*, *a7*, *b4*, and *b7*, were upregulated by hADSCs or SVF in mice skin, fibroblasts, and endotheliocytes.

Syndecans are transmembrane proteins on the plasma membrane that participate in cell signal transmission through multiple pathways [47]. Syndecan 1 is the core protein, with syndecan 4 found in the cytoplasm. Syndecan interacts with integrins through syndecans 1 and 4 and promotes integrin-mediated angiogenesis and cell attachment through an increase in Rho-mediated FAK phosphorylation [48]. In this study, we showed that syndecans 4, 1, and 2 are expressed at higher levels in fibroblasts and endotheliocytes than syndecan 3, where syndecans 1 (fold change: 2.49 in EC, 2.42 in Fs cells) and 3 (fold change: 2.42 in EC, 2.72 in Fs cells) expression significantly increased after treatment with hADSCs. Syndecan 2 and 4, on the other hand, increased to a lesser extent. As syndecan 1 not only has higher expression than the other syndecans, but also has a large increase in expression, suggests that syndecan 1 plays an important role in promoting wound healing.

Various metzincin enzymes participate in the activation of syndecan, and hydrolyzed fragments have biological activity. Studies have found that a variety of MMPs can digest syndecan to release active fragments in vitro and in vivo [49]. For example, activation of syndecans 1, 2, and 4 requires cleavage by the gelatinases MMPs 2 and 9 [50]. In this study, we found that *mmp9* mRNA expression increased, as measured by RNAseq and qRT-PCR, in fibroblasts and endotheliocytes after treatment by hADSCs.

Thrombospondins are a family of large multi-domain glycoproteins that regulate a range of cellular functions, including intracellular signals, migration, and proliferation, and they interact with a wide range of receptors, growth factors, proteases, and matrix molecules [43]. Thrombospondin 1 (*thbs1*) inhibits the migration and proliferation of endothelial cells and promotes apoptosis of endothelial cells by directly regulating CD36 and CD47 receptors, thus inhibiting angiogenesis [43, 51]. In this study, we found that *thbs1 and thbs3* mRNA expression decreased in endotheliocytes and fibroblasts treated by hADSCs. This downregulation of thrombospondins may promote angiogenesis.

Taken together, these data suggest that hADSCs promote endotheliocyte and fibroblast adhesion to ECM proteins, which is mediated by ECM-receptor interaction. At the same time, hADSCs promoted migration of fibroblasts and tubulogenesis properties of endotheliocytes and inhibited the contraction of local blood vessels, all of which cooperate to improve blood supply and angiogenesis during wound healing.

**Fig. 12:**
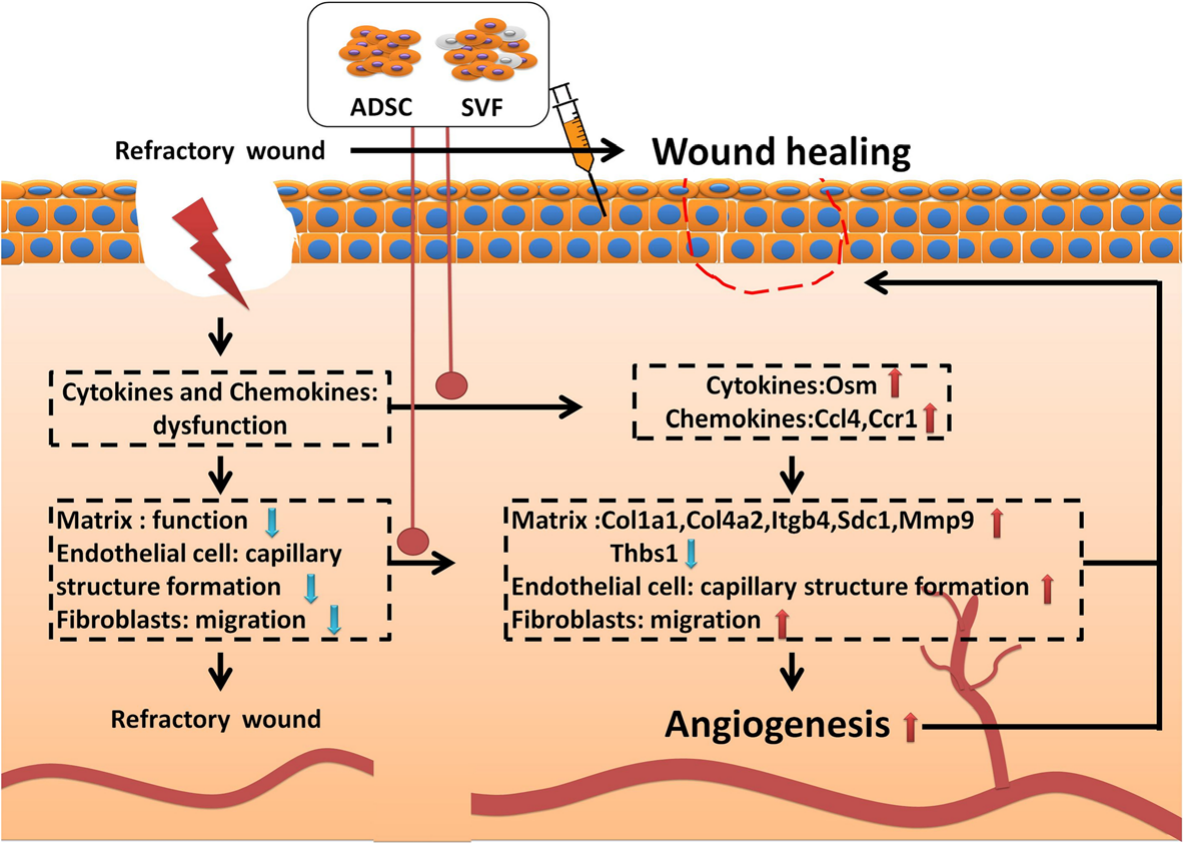
Proposed molecular mechanisms for improved wound healing by SVF. SVF promotes wound healing by inducing angiogenesis, matrix remodeling, and cell migration. Most processes are initiated through increased expression of chemokines and cytokines. Red arrows indicate the increase initiated by SVF or ADSCs. Blue arrows indicate the decrease

In summary, both SVF and hADSCs improve the function of endotheliocytes and fibroblasts, regulate gene expression, and jointly promote skin healing (Fig. 12). There is no significant difference in the effect, or mechanism, between SVF and hADSCs. Considering the convenience of SVF applications, SVF can replace hADSCs for wound healing.

## Conclusions

Stem cell therapy is a new research direction in medicine, and SVF-assisted treatment of wounds is a promising treatment. Various mechanisms are likely involved, including migration of fibroblasts, and tubulogenesis of endotheliocytes through the regulation of cell adhesion and the cytokine pathway. Since SVF affects multiple aspects of wound healing, we predict that interactions with surrounding cells and intricate paracrine activity play important roles in vivo. Further study of the interaction network of SVF and wound microenvironment is necessary and should provide a theoretical basis for the advancement of SVF-related cell therapy.

## Data Availability

The datasets used and/or analyzed during the current study are available from the corresponding author on reasonable request.

## Abbreviations

Angptl7: Angiopoietin-like 7
CCL: Chemokine (C-C motif) ligand
CCR: Chemokine receptor
Col: Collagen
EC: Endothelial cells
ECM: Extracellular matrix
Edn1: Endothelin1
FPKM: Fragments per kilobase of exon model per million reads mapped
Fs: Fibroblasts
hADSCs: Human adipose-derived stem cells
HE: Hematoxylin and eosin
hFs: Primary human fibroblasts
HUVEC: Human umbilical vein endothelial cell
Il: Interleukin
Itg: Integrins
Lam: Laminin
Lmod1: Leiomodin1
MSC: Mesenchymal stem cells
Osm: Oncostatin M
qRT-PCR: Quantitative reverse transcription polymerase chain reaction
STZ: Streptozocin
SVF: Stromal vascular fraction
Thbs: Thrombospondins
Vipr1: Vasoactive intestinal polypeptide receptor
